# A study on the construction of body fat percentage percentile curve for adults aged 20–79 in China

**DOI:** 10.3389/fpubh.2025.1598285

**Published:** 2025-06-20

**Authors:** Chunjing Tu, Qi Pan, Jinhui Zou, Lihong Liao, Xiaolong Chen, Yuyu Li, Xinzhe Pu, Yuanyuan Ding, Xiwen Luo

**Affiliations:** ^1^School of Teacher (Physical) Education, Taizhou University, Taizhou, China; ^2^School of Physical Education, Hangzhou Normal University, Hangzhou, China; ^3^Guangxi Zhuang Fang Autonomous Region Sports Science Research Institute, Guilin, China; ^4^Jilin Research Institute of Sports Science (Jilin Provincial Anti-Doping Center), Changchun, China; ^5^Xinjiang Uygur Autonomous Region Sports Science Research Institute, Wulumuqi, China

**Keywords:** GAMLSS model, body fat percentage, 20–79 years old middle-aged and older adult, percentile curve, critical value

## Abstract

**Objective:**

To establish age-specific Body Fat Percentage (BFP) percentile curves for Chinese adults aged 20–79 years, providing a reference tool for accurate BFP assessment and the prediction and prevention of obesity.

**Methods:**

Based on the data from 29,064 individuals obtained through the National Physical Fitness Surveillance (NPFS) in four provinces across China (East, South, West, and North), GAMLSS (Generalized Additive Models for Location Scale and Shape) was used to construct gender- and age- specific BFP percentile curves, and obesity cut-off points were defined.

**Results:**

First, age-specific BFP percentile curves were established for the P3, P5, P10, P25, P40, P50, P60, P75, P90, P95, P97 percentiles, and standardized Z-score curve for-3SD, −2SD, −SD, 0SD, +1SD, +2SD, and +3SD. Second, among Chinese adults aged 20–79 years, BFP increased with age before decreasing, with the peak occurring earlier for men than for women. The inflection points for men and women occurred at ages 65 and 73, respectively, with a difference of 8 years. Across all age groups, BFP percentiles for men were consistently lower than those for women. Third, the 75th percentile was identified as the cut-off point for BFP in defining obesity. The cut-off BFP for men across age groups ranged from 27.8–33.5%, and the cut-off for women ranged from 29.2–36.8%.

**Conclusion:**

The age-specific BFP percentile curves formulated in this study allow for the personalized and precise assessment of obesity in middle-aged and older adults. They also enable early prediction and risk warning of obesity in the older age groups. The findings support national goals such as “early intervention” and “precise assessment” and are recommended for use in practical settings to evaluate BFP and obesity in middle-aged and older adult populations in China.

## 1 Introduction

High Body Fat Percentage (BFP) has become an increasingly serious public health problem in China, where excessive BFP can lead to obesity. According to the World Obesity *Atlas* 2024 published by the World Obesity Federation, the adult obesity rate in China is rising at an annual rate of 2.8% (1). A 2019 cross-sectional study of 10.58 million Chinese adults found that 48.9% of subjects were overweight or obese, making obesity highly prevalent in China (2). Notably, China now has the world’s largest population of overweight and obese individuals (3). Although Body Mass Index (BMI) and BFP are widely used as indicators for assessing obesity, they cannot distinguish between fat mass and muscle mass (4), which may result in misclassification of individuals with increased fat mass and decreased skeletal muscle mass having a healthy weight, and those with high skeletal muscle mass but relatively low-fat mass as overweight and obese (5). In contrast, analyzing BFP through Bioelectrical Impedance Analysis (BIA) has been shown to be a more convenient, practical, and less invasive method for assessing body fat (6). BIA-derived BFP not only reflects body fat levels more accurately and intuitively but also enables precise evaluation of obesity severity.

High BFP seriously affects human health. Numerous cohort studies have shown that BFP directly affects insulin levels (7), and is positively correlated with insulin resistance (8), making it one of the important factors contributing to type 2 diabetes (9) excessive body fat promotes chronic systemic inflammation, which enhances cardiovascular metabolic risk and increases the incidence of cardiovascular disease (10). Obesity poses significant negative challenges to public healthcare system and population health. Controlling excessive BFP and reducing obesity prevalence are urgent public health issues that China needs to address. Therefore, it is crucial to establish more accurate BFP assessment and screening criteria for Chinese population, and to classify the corresponding obesity risk levels.

Currently, China lacks age-specific BFP standards for middle-aged and older adults. Age-based BFP percentile curves allow for precise evaluation of individuals, making it more scientifically valid than using the same standard across different age groups. Percentile curves have been widely applied in public health, such as in studies on children’s visual acuity and refractive changes (11), obesity rates and BMI variations (12), motor function measurements (13), and body composition indices (14). These studies mainly focused on children and adolescents, and have constructed percentile curves based on the Lambda-Median-Sigma (LMS) method or The Generalized Additive Model for Location, Scale, and Shape (GAMLSS) method. In 2020, the General Administration of Sport of China first incorporated BFP into the National Physical Fitness Surveillance (NPFS) system, marking the beginning of large-scale, population-based assessments of BFP in China. Therefore, before 2020, there was no widely representative percentile standard curve of BFP for middle-aged and older adult people aged 20 to 79 in China. After consulting the relevant literature after 2020, only the research on Wang Yuntao’s use of the percentile method to draw the distribution curve of body fat percentage in Guangdong Province was found (15). Consequently, it is of great value to establish a widely representative percentile standard curve of BFP in China.

In summary, this study uses a large sample of data from the NPFS across four provinces in China to construct age- and sex-specific BFP percentile curves for Chinese adults aged 20–79. The GAMLSS is employed to develop these curves, providing a reference tool for the diagnosis of obesity and preventing various obesity-induced diseases.

## 2 Research subjects and methods

### 2.1 Data sources

The study was conducted on the BFP of Chinese adults and older adults aged 20–79 years. The data were obtained from the 2020 NPFS program organized by the NPFS Center. The program covers four regions of China, including Zhejiang, Guangxi, Xinjiang and Jilin. The data were selected according to the principle of stratified random cluster sampling method. Considering the distribution characteristics of the data, each age group was divided into 5-years intervals. Using the standard range of [`X-3S, `X + 4S], data points for height and weight outside this range were excluded, as were those that did not meet the BFP standards. The final samples for modeling included 14,090 males and 14,975 females, with a total of 29,064 individuals. The sample sizes, means, and standard deviation for each age group are shown in Table 1.

**Table 1:** Mean and standard deviation of body fat percentage in the modeled population.

Age (year)	Male BFP (%)			Female BFP (%)		
	N	Mean	SD	N	Mean	SD
20–24	1,402	20.8	7.3	1,289	24.6	6.8
25–29	1,385	22.7	7.0	1,368	26.7	6.6
30–34	1,404	23.8	6.4	1,411	28.1	6.3
35–39	1,278	23.7	6.1	1,420	29.1	6.0
40–44	1,305	23.7	6.1	1,454	30.1	6.0
45–50	1,424	24.0	5.7	1,529	31.0	5.9
50–54	1,294	23.9	5.9	1,521	32.0	6.0
55–59	1,249	23.6	5.8	1,351	31.7	5.8
60–64	907	23.5	6.0	1,035	32.9	5.6
65–69	956	23.1	6.1	998	32.7	5.3
70–74	798	22.7	6.0	885	32.7	5.5
75–79	688	22.2	6.0	713	32.2	5.3
Total	14,090	23.2	6.3	14,974	30.1	6.5

### 2.2 Measurement method

The test method follows the relevant requirements of China’s NPFS Work Program formulated by the National Center for NPFS. The product quality of body fat percentage testing equipment must comply with the provisions of “General Requirements for National Physical Fitness Testing Equipment,” that is, the test instrument is a body fat analyzer with the following parameters: BIA method, featuring no less than 6 frequencies, with the highest frequency not lower than 1,000 kHz, and multi-segmental measurement capability. The test procedure is as follows: after the tester confirms that the instrument has entered the working state, the host screen inputs the basic information of the person’s gender and age. During the test, the subject removes shoes and socks barefoot, stands naturally on the foot electrode piece of the tester, holds the electrodes with both hands so that the thumb and palm are in contact with the electrodes, and the arms are separated from the torso by 15 degrees. The tester then initiates the test. The subject is instructed to maintains stability of the center of gravity, with body weight evenly distributed across both lower limbs, and to keep a quiet posture until the test is completed. The BFP result is recorded with an accuracy of one decimal place.

### 2.3 Research method

GAMLSS is used to construct age-specific BFP (BFP-for-age) percentile curve. SPSS software was used for preliminary processing of the eigenvalues of the sample data, and the modeling process was implemented through R-3.6.2 software.

## 3 Research process and results

### 3.1 BFP percentile curve construction

The main steps in the modeling process are as follows: firstly, select the optimal sub-model that can reflect the true nature of the original data, then adjust and optimize the curve of each parameter, and finally calculate the percentile curve and its reference value.

#### 3.1.1 PBF optimal sub-model and parameters

**Figure 1:**
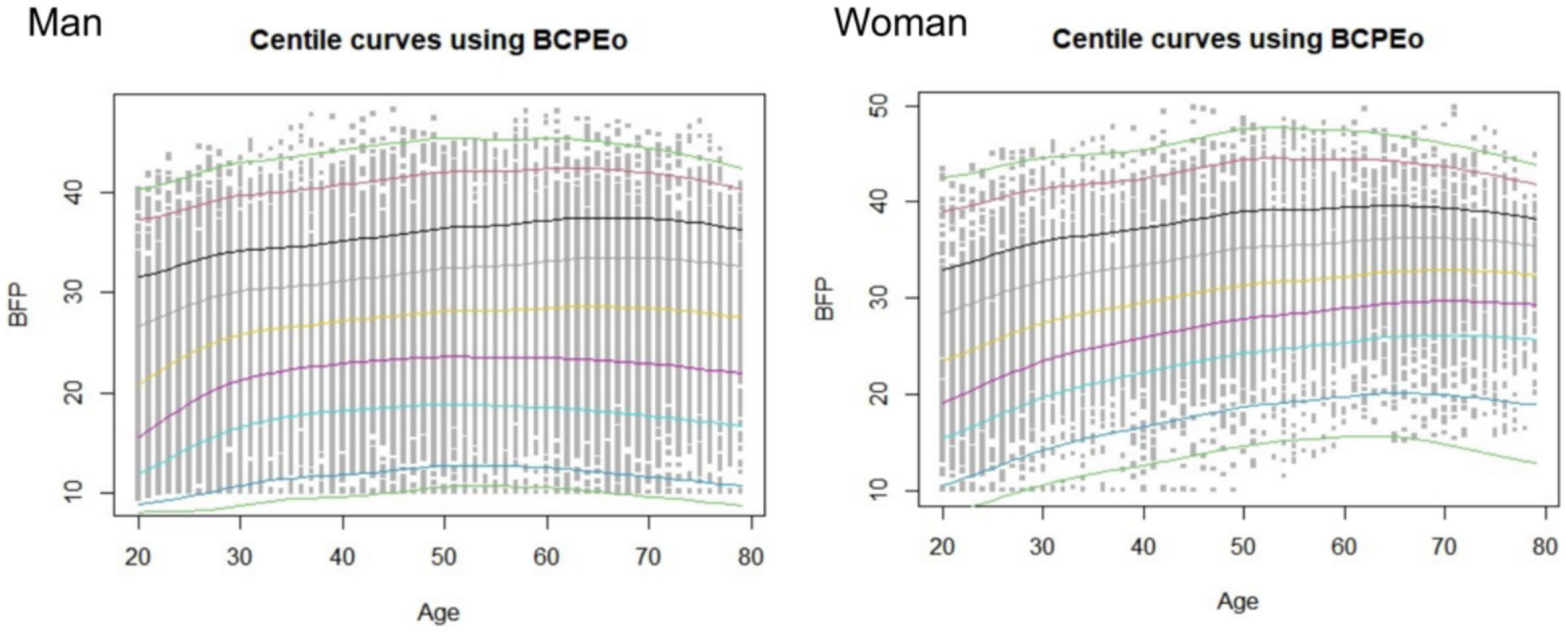
Initial fitting curve after iteration of LMS() function.

1. Selection of Optimal Sub-model. Based on the principles and methods of the GAMLSS model, model iteration was conducted using the LMS () function in R. The optimal sub-model was selected from among the GAMLSS sub-distribution models, such as BCCGo(*μ*,*σ*,*ν*,*τ*), BCTo(μ,σ,ν,τ), and BCPEo(μ,σ,ν,τ), etc. Since the sample size in this study exceeds n > 1,000, the Generalized Akaike Information Criteria (GAIC) and the Bayesian Information Criterion (SBC, also known as BIC) were used to determine the optimal model by selecting the smallest value. By comparing the minimum value of SBC for each sub-model, it was found that the optimal sub-model for male BFP was BCPEo, with the following values: GD (Global Deviance) = 195,054.8, AIC = 195111.5, and SBC = 195,346.3. When selecting the optimal model, the power transformation coefficient (*ξ*) for age and the initial degrees of freedom (df) of age were also obtained. The initial fitted percentile curves are shown in Figure 1.

#### 3.1.2 Curve fitting and optimization

The degrees of freedom of the parameters are crucial factors that determine the smoothness and goodness of fit of the curve. Parameters *μ*, *σ*, *ν*, *τ* were adjusted step-by-step for their initial degrees of freedom, and the iterative operation is performed by applying the gamlss() function. The Q-statistical test was applied to select the optimal curve parameters that balance both smoothness and goodness of fit. The process involved both quantitative and qualitative judgment, as outlined below.

**Figure 2:**
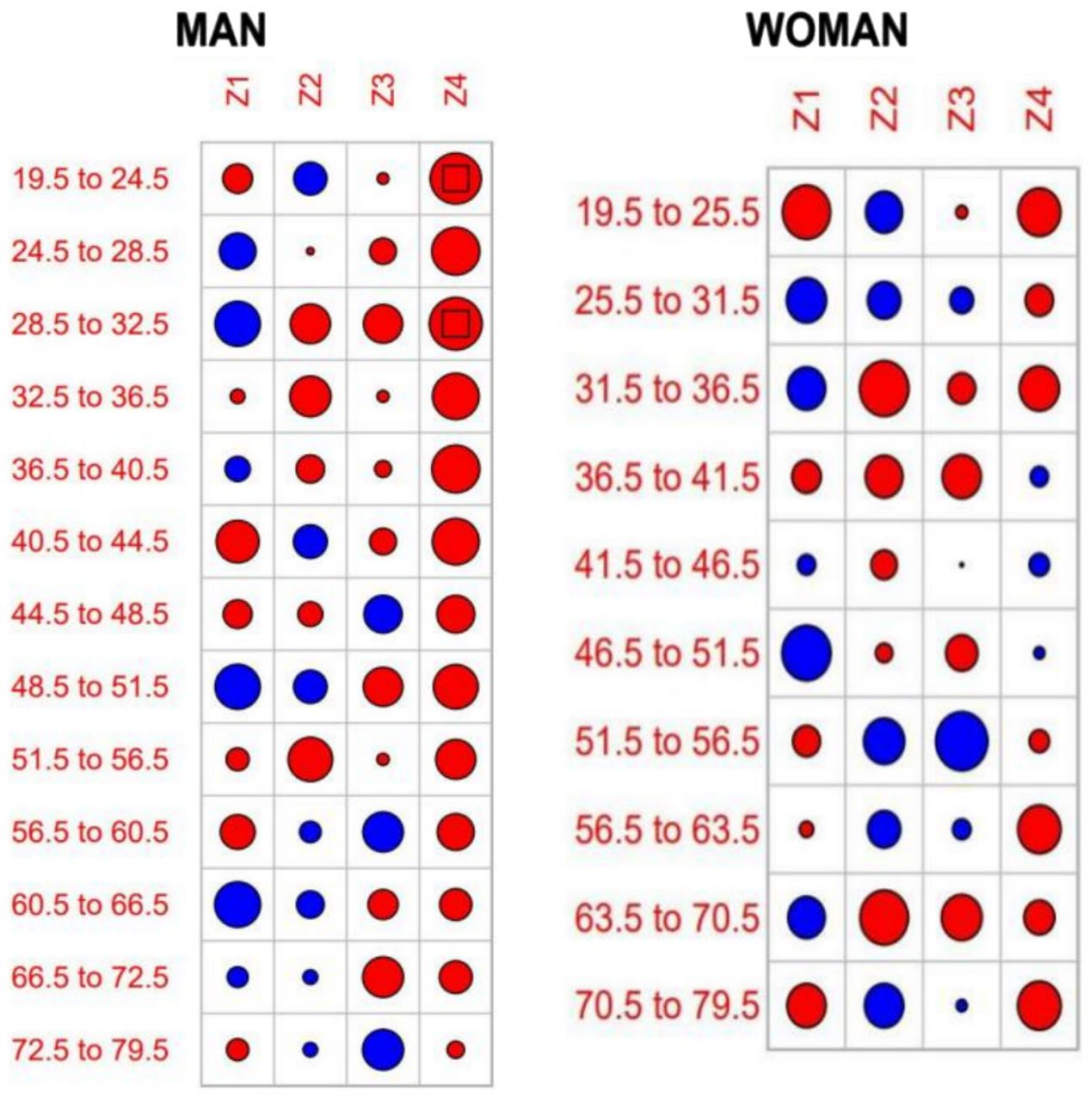
Q-statistical test plot of Z-quantile values for fitting model parameter residuals.

First, quantitative assessment (see Table 2): The criterion for evaluating the fitted curve parameter is the absolute value of Z should be less than 1.96 (*p* > 0.05). When a certain age group’s corresponding |Z_i_| ≥ 1.96 (i = 1, 2, 3, 4), the degrees of freedom of the corresponding parameter are incremented by 0.5 to re-model until |Z| value meets the criterion of being less than 1.96. If the individual |Zi| value slightly larger than the criterion but the overall Q-statistic *p* > 0.05, the result is still considered acceptable. In this study, the residual Z-values for the female parameters all met the criteria (*p* > 0.05). For males, the 19.5–24.5 years group and the 28.5–32.5 years group had |Z| values slightly larger than 1.96, but the overall Z-values met the criteria. Secondly, qualitative assessment (see Figure 2). The size of the circle in the figure is proportional to |Z| value, and the square at the center of the circle indicates |Z| > 1.96, suggesting potential mismatch. In this study, the Q-statistic circle plot showed that except for the 19.5–24.5 years group and 28.5–32.5 years age group for males (where the circles contained a square), all other age groups met the modeling requirements (*p* > 0.05), indicating good fitting results. Finally, the optimal models and parameters are obtained as follows: male, BCPEo (*ξ* = 0.41, df(*μ*) = 4.55, df(*σ*) = 2.50, df(*ν*) = 4.34, df(*τ*) = 8.98). Female, over comparison selection yields BCPEo (ξ = 0.87, df(*μ*) = 3.92, df(*σ*) = 1.38, df(*ν*) = 2.25, df(*τ*) = 1.12).

**Table 2:** Q-statistical test table for fitting Z-quantile values of model parameter residuals.

Age (year)	Male				Female			
	Z_1_	Z_2_	Z_3_	Z_4_	Z_1_	Z_2_	Z_3_	Z_4_
19.5–24.5	−0.66	0.82	−0.10	−1.98	−1.52	0.90	−0.09	−1.21
24.5–28.5	1.01	−0.04	−0.53	−1.75	1.05	0.73	0.36	−0.52
28.5–32.5	1.55	−1.21	−1.15	−2.10	0.96	−1.61	−0.50	−1.05
32.5–36.5	−0.16	−1.27	−0.11	−1.64	−0.57	−0.94	−1.00	0.21
36.5–40.5	0.48	−0.60	−0.21	−1.74	0.22	−0.45	−0.01	0.27
40.5–44.5	−1.38	0.86	−0.54	−1.65	1.58	−0.19	−0.68	0.08
44.5–48.5	−0.62	−0.46	1.09	−1.08	−0.50	1.10	1.80	−0.27
48.5–51.5	1.54	0.84	−1.17	−1.50	−0.13	0.72	0.22	−1.22
51.5–56.5	−0.40	−1.49	−0.11	−1.21	0.91	−1.55	−1.09	−0.62
56.5–60.5	−0.91	0.35	1.22	−1.02	−1.00	1.01	0.08	−1.24
60.5–66.5	1.59	0.57	−0.68	−0.77	−1.52	0.90	−0.09	−1.21
66.5–72.5	0.33	0.16	−1.25	−0.81	1.05	0.73	0.36	−0.52
72.5–79.5	−0.37	0.16	1.27	−0.22	0.96	−1.61	−0.50	−1.05
*p*-value of total	0.06	0.60	0.18	0.52	0.06	0.24	0.36	0.48

#### 3.1.3 Parameter curve fitting equations for distribution models

From the above parameters, the four parametric curve equations are obtained as follows, where cs() is the cubic spline function:

The parametric curve equations for *μ*, *σ*, *ν*, and *τ* related to male BFP are:

log(μ)=2.87+0.08685∗cs(Age^0.41,4.55).

log(σ)=−0.99−0.07335∗cs(Age^0.41,2.50).

ν=0.07+0.21856∗cs(Age^0.41,4.34).

log(τ)=0.49+0.05527∗cs(Age^0.41,8.98).

The parametric curve equations for μ, σ, ν, and τ related to female BFP are:

log(μ)=3.12–0.00982∗cs(Age^0.87,3.92).

log(σ)=−1.08–0.01893∗cs(Age^0.87,1.38).

ν=0.42+0.02244∗cs(Age^0.87,2.25).

log(τ)=0.75–0.00787∗cs(Age^0.87,1.12).

### 3.2 Percentile standard curve and standard Z-score reference values

**Figure 3:**
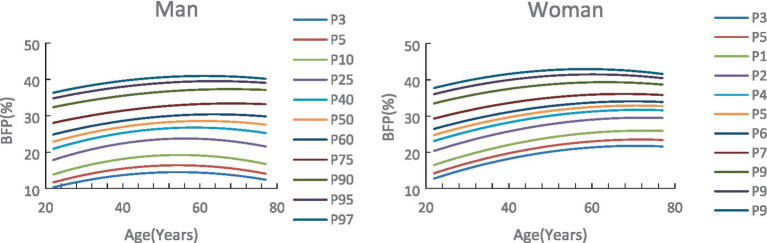
Body fat percentage percentile standardized.

Based on the above modeling process and results, the gender- and age-specific BFP percentile curves and standard Z-score curves were derived. The reference values for P_3_, P_5_, P_10_, P_25_, P_40_, P_50_, P_60_, P_75_, P_90_, P_95_, P_97_, and-3SD, -2SD, -SD, 0SD, +1SD, +2SD, +3SD are shown in Table 2 and Figure 3. The findings indicate that BFP for both males and females increased and then decreased with age, and there are gender differences. The reference values of the percentile curves for males are consistently lower than those for females across all age groups. P_50_ was further analyzed as follows (Table 2, Figures 2, 3):

1. Age trend characteristics: For males, the BFP increases with age from 20 to 64 years. The increase is rapid from ages 20 to 35 but slows down significantly from 35 to 64. After 65, BFP starts to decrease, and the rate of decrease accelerates with each passing year. For females, BFP shows a consistent increase from ages 20 to 69. Although the rate of increase remains moderate between ages 20 and 49, a continued upward trend is observed from 50 to 69 years, albeit at a slower pace. After 70, body fat percentage starts to decrease, and the rate of decrease increases during the 70 to 79 age range.
2. Gender comparison: the growth rates for males and females shift from positive to negative at ages 65 and 73, respectively. This means that males see their BFP change from increasing to decreasing eight years earlier than females. There is also a crossover in the growth rates between males and females at ages 29–30. Males experience higher growth rates than females from ages 20 to 29, but from ages 30 to 79, the growth rates for males are lower than those for females (see Figure 2) (Table 3).

**Table 3:** Reference value of percentile standard curve of BFP of Chinese 20–79 years old.

Age (year)	Reference values for percentile standard curves											Reference values for standardized Z-scores						
	P_3_	P_5_	P_10_	P_25_	P_40_	P_50_	P_60_	P_75_	P_90_	P_95_	P_97_	−3S	−2S	−1S	0S	+1S	+2S	+3S
Male																		
20–24	10.2	11.4	13.3	17.0	20.2	22.2	24.4	27.8	32.3	34.7	36.2	5.8	9.7	14.9	22.2	30.2	37.0	42.8
25–29	11.4	13.0	15.5	19.7	22.7	24.5	26.3	29.3	33.6	36.1	37.7	4.2	10.6	17.4	24.5	31.6	38.5	45.2
30–34	12.7	14.4	17.1	21.4	24.2	25.9	27.6	30.4	34.5	36.9	38.5	4.3	11.8	19.1	25.9	32.6	39.3	46.0
35–39	13.4	15.3	18.1	22.4	25.2	26.7	28.3	31.0	35.0	37.4	39.1	4.2	12.5	20.1	26.7	33.1	39.9	47.0
40–44	13.9	15.8	18.6	23.0	25.7	27.3	28.9	31.5	35.5	38.0	39.6	4.4	12.9	20.7	27.3	33.6	40.4	47.6
45–50	14.3	16.2	18.9	23.3	26.1	27.7	29.4	32.1	36.1	38.6	40.2	5.2	13.4	21.0	27.7	34.3	41.0	47.8
50–54	14.5	16.3	19.0	23.4	26.3	28.1	29.8	32.6	36.7	39.1	40.6	5.8	13.6	21.0	28.1	34.8	41.4	47.9
55–59	14.4	16.2	18.9	23.4	26.4	28.2	30.0	32.9	37.0	39.4	41.0	5.5	13.4	21.0	28.2	35.2	41.7	48.1
60–64	14.2	15.9	18.6	23.2	26.4	28.4	30.3	33.4	37.4	39.7	41.1	5.6	13.3	20.7	28.4	35.6	41.8	47.4
65–69	13.7	15.5	18.2	22.9	26.3	28.3	30.4	33.5	37.5	39.6	40.9	5.4	12.9	20.3	28.3	35.7	41.6	46.5
70–74	13.2	15.0	17.7	22.5	26.0	28.1	30.2	33.4	37.3	39.3	40.6	4.8	12.3	19.9	28.1	35.6	41.2	45.7
75–79	12.6	14.4	17.2	22.0	25.6	27.8	29.9	33.1	36.9	38.9	40.0	4.2	11.7	19.4	27.8	35.2	40.6	44.8
Female																		
20–24	12.8	14.2	16.5	20.3	23.0	24.7	26.4	29.2	33.5	36.1	37.8	6.6	12.1	18.2	24.7	31.5	38.7	46.1
25–29	14.5	16.0	18.3	22.1	24.8	26.4	28.0	30.8	34.9	37.5	39.1	7.7	13.8	20.1	26.4	33.0	40.0	47.5
30–34	16.1	17.6	19.9	23.8	26.4	27.9	29.4	32.1	36.1	38.6	40.2	8.8	15.3	21.7	27.9	34.2	41.1	48.5
35–39	17.4	19.0	21.3	25.1	27.6	29.1	30.5	33.1	36.9	39.4	41.0	9.8	16.6	23.1	29.1	35.1	41.8	49.2
40–44	18.6	20.1	22.5	26.2	28.7	30.1	31.5	33.9	37.7	40.1	41.7	10.7	17.8	24.3	30.1	35.9	42.5	49.8
45–50	19.7	21.2	23.6	27.2	29.6	31.0	32.3	34.7	38.4	40.8	42.4	11.8	18.8	25.3	31.0	36.6	43.2	50.6
50–54	20.5	22.1	24.4	28.0	30.3	31.7	33.0	35.3	38.9	41.3	42.9	12.6	19.7	26.1	31.7	37.2	43.7	51.0
55–59	21.1	22.7	25.0	28.6	30.9	32.1	33.4	35.7	39.2	41.5	43.0	13.0	20.3	26.7	32.1	37.5	43.8	50.8
60–64	21.6	23.2	25.6	29.1	31.4	32.6	33.8	36.0	39.4	41.6	43.0	12.9	20.7	27.3	32.6	37.8	43.8	50.4
65–69	21.8	23.5	25.9	29.5	31.7	32.9	34.1	36.2	39.4	41.4	42.8	12.3	20.9	27.6	32.9	37.9	43.5	49.5
70–74	21.7	23.5	26.0	29.6	31.7	32.9	34.1	36.1	39.1	41.0	42.2	11.3	20.8	27.7	32.9	37.7	42.9	48.3
75–79	21.6	23.4	25.9	29.5	31.6	32.8	33.9	35.8	38.6	40.4	41.5	10.3	20.6	27.7	32.8	37.3	42.1	47.0

## 4 Discussion

This study utilizes data gathered from the NPFS conducted across four provinces in southeastern, northwestern, and northern China. The GAMLSS method was employed to construct age- and gender-specific percentile standardized curves for BFP among adults aged 20 to 79 years. These curves serve as a reliable reference tool for accurately assessing and predicting obesity risk, providing valuable insights for early intervention. This approach is vital for addressing the rising obesity epidemic and its associated health risks.

Characterization of age trends in body fat percentage in China. The study results indicated that BFP in Chinese middle-aged and older adult individuals aged 20 to 79 years initially increased and then decreased with age, with significant gender differences. Regarding age trends, the study found that the obesity rate of Chinese middle-aged and older adult people was at a high level. The growth rates of men and women turned from positive to negative at 65 and 73 years of age, respectively. Men experienced a transition from increasing to decreasing body fat 8 years earlier than women. A study of adult BFP in Japan show (16) that the BFP of Japanese men reaches its peak at the age of 70 to 79, while that of women reaches its peak at the age of 50 to 60. The male inflection point occurred earlier in China than in Japan, while the female inflection point occurred later in China than in Japan. The reasons for this difference are closely linked to lifestyle, physical activity, dietary habits, and environment factors. The accelerated urbanization has led to a more sedentary lifestyle in China, with physical activity-related work and travel being replaced by machinery and equipment. It might also be related to the fact that the Japanese study was conducted earlier, leading to differences in findings. This transition has resulted in a significant reduction in the amount of time spent in physical activity. In addition, higher work stress often leads to irregular eating and sleep patterns, which can increase appetite (17), thus increasing the risk of obesity. Poor sleep quality reduces adiponectin levels, which are negatively associated with obesity (18). In terms of diet, results from a prospective cohort study of UK adults showed that consumption of a diet rich in ultra-processed foods increased the risk of obesity by 79% and abdominal obesity by 30% (19). Other potential mechanisms for the link between ultra-processed diets and obesity may be related to their low satiety and induction of a hyperglycemic response (20). Furthermore, environmental factors also have multiple potential pathways of influence on obesity. Research on air pollution in China has shown that fine particulate matter (PM_2.5_), and lower air quality are significant correlates of obesity (21).

Regarding gender differences. The BFP of women is higher than that of men at all ages, and this gap gradually widens with age. The peak BFP in women occurs 8 years later than in men. The reasons for this may be related to the differences in physiological conditions between men and women, the social division of labor, and technological progress. Physiologically, women are affected by the decline of hormone levels after menopause, and the risk of obesity increases (22). From a societal perspective, in the social division of labor, society is more inclined to allocate or choose women to undertake work with a lower degree of physical labor. Furthermore, the widespread adoption of labor-saving technologies in the home—such as automated appliances—has significantly reduced the amount of physical activity required for domestic tasks, contributing to decreased energy expenditure among women.

Application of findings to the development of an obesity cut-off point. Pawel Macek et al. (23) suggest that the 75th percentile could serve as the cut-off for BFP to determine obesity. In this study, the BFP cut-off values for Chinese men of all age groups ranged from 27.8 to 33.5%, and the total cut-off average for all ages was 31.2%. For women, the cut-off values ranged from 29.2 to 36.8%, with an overall mean of 34.1%. World Health Organization (WHO) has not established a percentile curve for adult body fat percentage, nor has it divided the cut-off points for body fat percentage at different ages, WHO recommends that adult males are classified as obese if their BFP exceeds 25%, while for females, the threshold is 35%. In this study, the cut-off point for males is 6.2 percentage points higher than the WHO recommendation and 0.9 percentage points lower for females. In contrast, a study by Fan et al. (24), which used ROC curves analysis, reported obesity cut-off values for the Chinese adults aged 18–69, with male and female BFP cut-offs of 23.2 and 36.4%, respectively. The male cut-off in this study is 8.0 percentage points higher, while the female cut-off is 5.2 percentage points lower. These differences may be attributed to variations in the study populations, as the previous studies did not establish age-specific cut-off standards. BFP demonstrates complex associations with genetic predisposition, psychosocial factors, environmental influences, and individual health behaviors. Moreover, substantial variations in body composition patterns exist across sex and age groups (25). Age-related changes in body composition typically manifest as decreased muscle mass and increased adiposity (5). Application of uniform BFP criteria across all age groups may increase the risk of misdiagnosis and under diagnosis in obesity screening. Consequently, given the age-dependent variability of BFP, age-stratified thresholds should be established. The BFP thresholds derived from this study demonstrate scientific validity for obesity identification. In clinical practice, an integrated assessment incorporating BFP, BMI, and other adiposity indicators is recommended to provide a comprehensive evaluation of obesity.

### 4.1 Limitations

The BFP testing equipment used in this study is “General Requirements for National Physical Fitness Testing Equipment.” However, if health professionals do not have access to such equipment when using the tabulated data from this study to evaluate health status, the applicability of the study’s results may be limited.

## 5 Conclusion

BFP is commonly used to assess obesity in clinical, research, and community settings. This study utilized national survey data from four provinces in southeast, northwest, and northern China to establish gender- and age-specific BFP percentile standard curves. It also defined the cut-offs for different age groups. The findings from this study can be applied in various ways: they can facilitate comparisons of body fat percentage within the same gender and age group, be used for trend analysis based on age, and help in predicting and warning against potential obesity risks. Additionally, this tool can be instrumental in monitoring healthy aging, contributing to the improvement of national physical fitness and health levels.

## Data Availability

The raw data supporting the conclusions of this article will be made available by the authors, without undue reservation.
